# Daily bread: a novel vehicle for dissemination and evaluation of psychological first aid for families exposed to armed conflict in Syria

**DOI:** 10.1017/gmh.2016.9

**Published:** 2016-04-22

**Authors:** A. El-Khani, K. Cartwright, A. Redmond, R. Calam

**Affiliations:** The University of Manchester, Manchester, UK

**Keywords:** Caregiver, conflict, displacement, interventions, parenting support, refugee

## Abstract

**Background.:**

Risks to the mental health of children and families exposed to conflict in Syria are of such magnitude that research identifying how best to deliver psychological first aid is urgently required. This study tested the feasibility of a novel approach to large-scale distribution of information and data collection.

**Methods.:**

Routine humanitarian deliveries of bread by a bakery run by a non-governmental organisation (NGO) were used to distribute parenting information leaflets and questionnaires to adults looking after children in conflict zones inside Syria. Study materials were emailed to a project worker in Turkey. Leaflets and questionnaires requesting feedback were transported alongside supplies to a bakery in Syria, and then packed with flatbreads. Three thousand bread-packs were distributed, from three distribution points to which questionnaires were returned, and then taken to Turkey and dispatched to the UK.

**Findings.:**

Notwithstanding delays, 3000 leaflets and questionnaires were successfully distributed over 2 days. Questionnaire return yielded 1783 responses, a 59.5% return rate. Overall ratings of the usefulness of the leaflet were 1060 (59.5%) ‘quite a lot’ and 339 (19.0%) ‘a great deal’. Content analysis was used to code 400 respondent comments. Four themes emerged; positive comments about the leaflet, suggestions for modifications, descriptions of children's needs and the value respondents placed on faith.

**Interpretation.:**

Findings indicate the willingness of NGO staff and volunteers to assist in research, the remarkable willingness of caregivers to respond and the value of brief advice. It demonstrates the scope for using existing humanitarian routes to distribute information and receive feedback even in high-risk settings.

## Background

The Syrian crisis has brought to the forefront the enormous challenges that families face in the context of war and displacement. To date, over 7 million people are internally displaced; at least half of those internally and externally displaced are children (UNHCR, 2013). Studies suggest that the mental health of Syrians that have been displaced indicate rising levels of psychological distress, this is in the context of minimal mental health and psychosocial support services for internally displaced people and refugees in difficult to access areas (Abou-Saleh & Hughes, 2015). In immediate humanitarian crises, the focus is on shelter, food and essential medical care. However, loss and adversity, disruption and adaptation to new environments pose additional significant risks to mental health (Drury & Williams, 2012). Building resilience and optimising mental health is fundamental to longer term adjustment, and reducing emotional suffering and promoting mental health is therefore a major global health challenge (UNICEF, 2004; Patel *et al*. 2007, 2008). Systematic reviews show that the key protective factors for refugee children include settling in a stable context with social support, parental support and family cohesion, perceived support from friends and good experiences in school (Fazel *et al*. 2012). A review of preventive interventions for children exposed to armed conflict, including refugees, noted the paucity of high-quality research on interventions in these contexts and the need for ‘psychological first aid’ to be embedded into programmes in primary health and education (Peltonen & Punamäki, 2010). The scale of the Syrian crisis makes plain the impossibility of providing individual interventions for all families at risk of mental health difficulties. Given the scale of the problem, one priority for delivery of psychosocial interventions in this context is to identify ways of providing information at the population level as part of a public health model (Mollica *et al*. 2004) and evaluating these.

Families represent the front line of defence for children's mental health. Promoting preventive approaches which provide information tailored to the community and context to help families provide warm, supportive parenting is one means of offering a relatively low-cost method for strengthening support for children. However, building the evidence base for helpful interventions in highly risky settings requires a feasible means of obtaining data.

We conducted pilot work on parenting in refugee camps in Turkey and Syria, including focus groups and interviews with 27 parents and two non-governmental organisation (NGO) staff in the field (El-Khani, 2015). Questionnaires assessing the mental health of children were also administered to 106 parents and caregivers (Cartwright *et al*. 2015). This work revealed that very soon after the immediate extreme stress of displacement, parents were very keen to access information on how best to parent their children in this new context and were making active attempts at reaching support from NGO workers, health professionals in the refugee camps as well as seeking advice from other parents. They willingly completed brief questionnaires and participated in focus groups, where they talked constructively about their needs. Parents made plain their need for information on how best to care for their children in this context. The pilot work made clear the potential advantages of families having access to information to support them in their parenting struggles. Working with a NGO, Watan, many hours were spent in discussions as to how we could reach families at a population level and low cost. We identified several possible means, such as reaching families via refugee camp schools or aid distribution points. Watan suggested that we could distribute printed material rapidly to very large numbers of identified families in need alongside their routine distribution of bread via their humanitarian assistance charity, Khayr Charity Foundation, and obtain data on parent and carer views through this means. This method suggested the greatest potential means of rapid distribution of information and data collection. The aim of the study was to test the feasibility of the bread wrapper approach; firstly, to distribute information to families [including both internally displaced persons (IDPs) and existing inhabitants] living inside Syria and secondly, to obtain completed questionnaires from families via the same bakery distribution routes. The questionnaires enabled parent feedback on the perceived usefulness of information provided. As this approach was entirely novel, a check was made on whether there were any significant differences in how useful IDP's and existing inhabitants found the leaflet.

## Methods

Routine daily delivery of bread from a bakery in Syria run by Khayr was used to distribute parenting information leaflets and questionnaires to parents and other caregivers living with children in a conflict zone in Northern Syria close to the Turkish border. At the time, families in the area surrounding the bakery comprised 60% IDPs and 40% existing inhabitants. For many of the IDPs this was often their second and sometimes third relocation point since fleeing their homes.

Every individual listed as living within 10 km of the bakery received bread routinely regardless of whether they were an IDP or existing resident. Bread distribution was carried out daily by approximately 200 volunteers using frequently updated lists for streets and blocks of housing which included parent/caregiver status and needs, and thus households with children could be identified, allowing distribution of study materials to appropriate recipients.

**Fig. 1.**
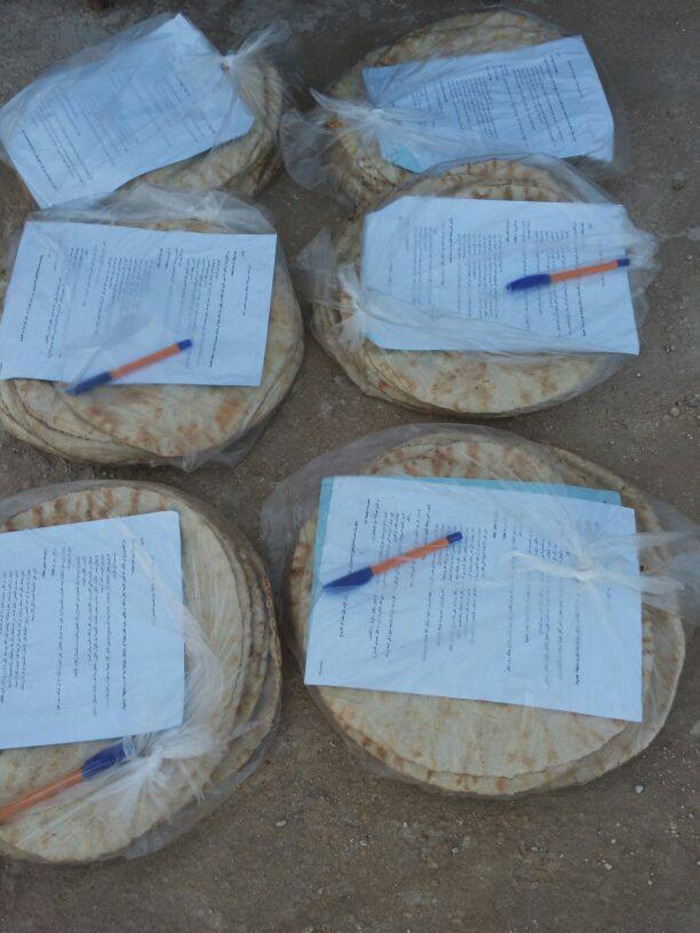
Study materials packed in flat breads.

A local project worker was identified by Watan to manage the study from Turkey. Study materials and a very detailed, specific research protocol were emailed to the project worker in Turkey, where parenting information leaflets and questionnaires were printed and subsequently transported alongside bakery supplies to the bakery in Syria. A field officer led the study from the bakery inside Syria and co-ordinated volunteers who assisted in packing and distributing materials there. The bakery was 130 km from the Bab al-Hawa Border Crossing, an international border crossing between Syria and Turkey. At the bakery, questionnaires, numbered and colour coded to enable data tracking across different distribution points, plus pens, were packed inside transparent plastic bags containing the daily provision of flatbreads (Fig. 1). Three thousand bags of bread enclosing study materials were distributed to families listed as including an adult caregiver of a child or children from three of the bakery's surrounding distribution points. These were located within 3 km of the bakery to the north and east. The field officer and volunteers then supervised the return of questionnaires into boxes at the distribution points and then to the bakery over a period of 5 days.

Questionnaires were taken back to Turkey over 5 days where they were photocopied to prevent data loss and despatched 10 days later to the UK. Photocopies held in Turkey were destroyed confidentially following receipt of the original questionnaires in the UK. All stages of the study were photographed to verify activity. ‘WhatsApp’©, which allows photographs to be shared, and, when available, email and ‘Skype’ © were used to monitor the progress of the study. Free text comments written onto questionnaires were translated by the lead author who is bilingual English/Arabic.

Leaflets were drawn from a range of sources, informed by: (1) prior qualitative work with families and practitioners in refugee camps in Turkey and Syria; (2) Syrian refuges resettled in the UK (El-Khani, 2015); (3) relevant information from a range of NGO's available online; and (4) literature on key components of parenting interventions (Kaminski *et al*. 2008). The leaflet comprised two sides of A4 with four sections: (1) what caregivers might be experiencing; (2) what children might be experiencing; (3) how caregivers can help themselves; and (4) how caregivers can help their children (including safety, providing warmth and support, giving praise, spending time together and talking; encouraging play and maintaining routine). The leaflet was reviewed several times by both Watan workers as well as a Syrian refugee advisory group in Manchester, before the leaflet was finalised. The leaflet and further information is available online. http://sites.psych-sci.manchester.ac.uk/pfrg/resources/ Questionnaires covered whether, and how long, families had been displaced, basic demographics, who caregivers talked to about parenting concerns, and then asked specific questions about the leaflet, including an overall rating, and ratings of each of the four sections of the leaflet on a four point scale, ‘not at all useful’ to ‘a great deal’. Space for comments was provided. All materials were translated into Arabic with back translation to ensure fidelity.

Approval was obtained from The University of Manchester's Research Ethics Committee. In addition the authors assert that all procedures contributing to this work comply with the ethical standards of the relevant national and institutional committees on human experimentation and with the Helsinki Declaration of 1975, as revised in 2008. The protocol and risk assessment was developed with and reviewed by Watan and Khayr. The University of Manchester Humanitarian Conflict Response Institute and University ethics committee were consulted throughout. Some of the ethical considerations of working in this context are described separately (El-Khani *et al*. 2013).

## Results

Parenting information leaflets and questionnaires were successfully distributed over 2 days to 3000 parents and caregivers in total, 1500 from the first bread distribution point, (A), 1000 from the second (B) and 500 from the third (C). The field officer and volunteers were able to distribute packs and obtained completed questionnaires with no adverse events. The return rate overall was 1783, 59.5%, comprising 740 (49.3%) for location A, 690 (69%), location B and 354 (71%) for location C. In addition, 400 respondents wrote comments on the questionnaire.

Table 1 shows the sample characteristics of the respondents; 1271 (71.3%) were internally displaced and 492 (27.6%) existing residents, [missing data 20 (1.1%)].

**Table 1.** Sample characteristics

Sample characteristics	Frequency (*n* = 1783)	Percentage
Gender		
Male	1307	73.3
Female	452	25.4
Missing	24	1.3
Number of children		
0	5	0.3
1–4	1010	56.6
5–8	714	40.0
9–12	32	1.8
Missing	22	1.2
Internally displaced		
Yes	1271	71.3
No	492	27.6
Missing	20	1.1
Length of time in current location (for displaced families)		
<3 months	373	29.3
3–6 months	404	31.8
6–12 months	199	15.7
>12 months	278	21.9
Missing	17	1.3

The majority of respondents reported seeking parenting support (1719, 96.4%) (Table 2).

**Table 2.** Who caregivers seek parenting support from

Variable	Frequency (*n* = 1783)	Percentage
Support sought for parenting issues		
Yes	1719	96.4
No	60	3.4
Missing	4	0.2
Who caregivers seek parenting support from		
Parent	676	39.3
Friend	579	33.7
Health worker	450	26.2
Sibling	441	25.7
Religious leader	398	23.2
Neighbour	373	21.7
Grandparent	331	19.3
School teacher	307	17.9
Charity worker	297	17.3
Aunt	210	12.2
Cousin	160	9.3
Uncle	150	8.7

**Fig. 2.**
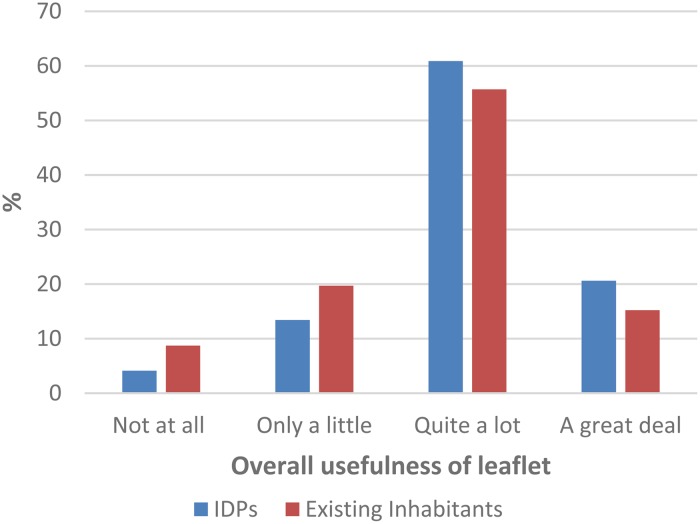
Perceived overall usefulness of parenting leaflet.

Overall, the majority of families, both IDPs and existing residents rated the usefulness of the leaflet (Fig. 2) as ‘quite a lot’ [IDPs: 774 (60.9%), existing residents, 274 (55.7%)] or ‘a great deal’ [IDPs: 262 (20.6%), existing, 75 (15.2%)].

The *t* tests indicated a significant difference on all five usefulness questions between IDPs and existing residents, with IDPs giving higher ratings (Table 3).

**Table 3.** Mean ratings for perceived usefulness of the advice on the leaflet, IDPs and existing residents

	Overall means (*n* = 1783)		Internally displaced 1271 (72.1%)		Existing residents 492 (27.9%)		*t*
	Mean	s.d.	Mean	s.d.	Mean	s.d.	
Overall usefulness	1.93	0.75	1.99	0.71	1.78	0.81	−5.06***
What might you be experiencing	2.02	0.79	2.08	0.76	1.86	0.88	−4.77***
What can you do to help yourself	1.92	0.83	1.96	0.81	1.84	0.87	−2.44*
What might your child be experiencing	2.04	0.81	2.09	0.77	1.93	0.88	−3.47**
What can you do to help your child	2.06	0.82	2.10	0.79	1.95	0.88	−3.36**

Scale: 0, not at all to 3, a great deal.

*p < 0.05, **p < 0.01, ***p < 0.001.

Respondent comments were coded using a bottom-up, multi-stage content analysis process. Inter-rater reliability was ensured by a second researcher independently coding the data set and then a third researcher comparing and synthesising the two data sets into one. Four main themes emerged; positive comments about the leaflet, suggestions for modifications, a description of their children's needs and the value respondents placed on their faith (Table 4). Nineteen comments were coded twice into two separate themes.

**Table 4.** Frequency of comments coded in themes *(*n = 400*)*

	Frequency	Percentage
Theme 1: positive comments	289	68.97
Theme 2: modifications suggested	67	15.99
Theme 3: needs	36	8.59
Theme 4: faith	16	3.82
Excluded	11	2.63

The highest numbers of comments were in the positive comment theme. Respondents praised the content of the leaflet for usefulness and clarity. One caregiver wrote ‘I have been waiting for something useful like this after not finding anyone to answer my questions’. Another wrote ‘This is great if we follow it accordingly. It has relaxed us and shown us what to do. We can reduce anxiety and fears in our children and make them feel safer’. Messages described increased positivity, motivation and caregiver self-confidence on receiving the leaflet.

Caregivers left comments suggesting what modifications they felt needed making to the leaflet. They suggested they wanted more detailed information on emotional difficulties such as anxiety and dealing with bereavement. One caregiver wrote ‘I wish there was more attention and details on how to deal with fear; our children are really suffering from this’.

Caregivers described both children's physical needs (such as clothing, medicine and food) and psychological needs. One caregiver wrote ‘The children are complaining a lot now of physical problems and the main reasons for this is the stress they are facing from how fearful they are during these really challenging times’. High levels of fear, bedwetting and anxiety were the most common psychological problems reported. Finally, caregivers' comments contained references and supplications to God such as ‘We turn to God for support’. Some comments were only prayers or reference to god, while others ended with a religious supplication.

## Discussion

This study demonstrates the feasibility of a means of both distributing information and receiving responses which fitted readily into existing humanitarian frameworks. The efficiency with which the NGO distributed 3000 leaflets and questionnaires in 2 days and the very high return rate of 59.5% from families exemplifies the potential that this approach offers for rapid dissemination of information to families and for families to give feedback. To our knowledge this is the first time this approach has been used. We received photographic reports via ‘WhatsApp’© on the progress of the study from the printing of materials in Turkey, through their wait for several days in the bread supply trucks on the Syrian border, their distribution packed into the supplies of bread for each family, to their return. Via Skype ©, we heard of the cheers of people queuing at the bakery distribution points when parents dropped off completed questionnaires. While we do not have exact data on the literacy rates of families who were involved in the study, we were informed by our field officer that these were high. Often if one member of the family was illiterate there was another member in the family who could read and write. We received a photograph of a child completing the questionnaire on behalf of his mother who could not read or write, a valuable observation for planning the framing and presentation of future resources.

We were open minded over how valuable caregivers in this context would find the leaflet, but were pleased to find that 81.5% of IDP caregivers rated its overall usefulness as either ‘quite a lot’ or ‘a great deal’. In addition, of the five items caregivers were asked to rate, three were rated as ‘quite a lot’ or more. These results indicate the potential for brief written material to assist families in better caring for their children in the challenging environment. The comments left by caregivers further supported the usefulness of the leaflet, with 69% of the 400 comments being positive. Several comments expressed thoughts and ideas that were not mentioned in the leaflet such as ‘giving yourself a chance to relax emotionally is very important so that you can take control of your actions and encourage good behaviours in your children’. It is possible that the leaflet may have prompted parents to think and reflect on their parenting and what strategies they could adopt to better care for their children. Similar findings were found in our previous exploratory work with Syrian refugees; simply taking part in conversations about parenting challenges led parents to reflect on how they could improve and adapt the approaches they were using with their children (El-Khani, 2015). Parents wrote very useful and detailed comments too on what modifications they felt were necessary to improve the leaflet. Not only is this information very important for future replication and dissemination; it also provided further insight into what key difficulties caregivers were struggling with. We had included analysis to identify whether there were any differences between IDP's and longer standing residents. It is noteworthy, but perhaps not surprising, that the families who had been displaced found the information significantly more helpful. These were families experiencing high levels of difficulty, having had to abandon their homes, and the stress of family life in displacement would be expected to be higher, putting strain on parenting resources.

Of paramount concern was the safety of all staff and families. Detailed protocols were designed to cover anticipated risks. Tragically, one bakery we had planned to work with was bombed before the study commenced. The area was deemed too dangerous for UK researchers to travel to, and the study was therefore run remotely by local staff already in place and well versed in management of day-to-day security. We were conscious of the risks posed if materials were not acceptable, and of the security of the data itself. At the NGO's request, the parenting information leaflets and questionnaires carried no identifying information regarding their source. Returned questionnaires were photocopied as quickly as possible following collection and crossing the border to Turkey and kept separately in case of loss of the original versions during transport to the UK. In presenting the data, actual locations remain confidential. Since this study has been completed, heavy air strikes in the area mean many of the existing families have now left for Turkey and new families have arrived from other parts of Syria, highlighting the struggle and often constant movement refugees experience in search of safety and the need for support to be accessed wherever they are.

The field officer highlighted to us the significance of the relationship and trust we had built with the NGO Watan and its advantages for the study. We had worked closely with Watan over the past 2 years on background exploratory research; therefore they trusted our team's expertise and research motivation and were eager to support this study. Likewise, we trusted their capability to follow our ethical requirements and execute the study professionally. On the ground, the families engaged in the study and returned questionnaires as they were distributed from a source they trusted. With such vast numbers of bread packs being delivered daily, many of those involved in bread distribution were members of the local community who were volunteering their time. We had no indications that families were concerned that their confidentiality would be compromised. These cycles of trust were paramount.

A study carried out under these conditions and with a very limited budget has many limitations.

It was essential to keep the feedback questionnaire brief and not to exceed one double sided sheet of paper, in order to ensure low costs and maintain information integrity which could be at risk through stapling multiple sheets, therefore there are many questions that we could not explore, such as if those that completed the questionnaires were parents or caregivers to the children or how long these children had been under their care. Also, although the majority of those that completed the questionnaires indicated that they were male (73.3 %), it is possible that this is not an actual reflection of the responses. Volunteers informed the research team that many fathers indicated, during the return of the questionnaires, that responses were actually informed by their wives, but that they had indicated ‘male’ as they had returned the questionnaires. The feedback questionnaires indicated that confidentiality will be maintained at all times and no names or identifying materials were requested. Though there was no indication that families were concerned about confidentiality, as they had not raised any concerns with volunteers or NGO workers, we cannot be sure that some may not have returned the questionnaires because of this, especially in the highly volatile political time that this study was conducted. In addition, under more secure circumstances, it would have been excellent to test whether the leaflet actually brought about any changes in family life, over and above the boost to morale so evident in the comments provided by families.

The demonstration, on a large scale, of the feasibility of this effective, family-specific communication and research channel helps to establish a basis for the further development and testing of materials to provide psychological support to families which can act as the base layer of a public health approach in a complex, changing context, where very little psychological assistance is available (Peltonen & Punamäki, 2010). Peltonen and Punamaki identify ‘new generation’ preventive interventions which start from recognition of strengths and vulnerabilities of specific groups and work to enhance existing protective mechanisms and elements that promote healing and resilience, attending to culturally salient, appreciated, traditional ways of addressing distress. The development and evaluation of culturally appropriate and evidence-based materials, which can rapidly be made widely available for distribution, is essential. We did not undertake formal cost effectiveness analysis, but the overall cost of local printing, distribution and collection of questionnaires over and above the NGO's pre-existing costs for bread distribution was approximately 18 UK pence (25 cents US) per family. This indicates the scope for approaches using existing humanitarian supply routes to distribute information across a wide range of contexts and emergencies of all kinds. The value of this kind of piggy-backing merits much more extensive investigation across low resource settings. We have placed the materials developed in the course of the study online to allow open access, with some translations of leaflets into other languages. The leaflet has already been used, with minor modifications, for newly arrived families in Sweden. Evaluating the effects of providing this kind of information is now needed.

Evaluating the outcome of interventions in conflict zones is challenging; the leaflets were designed to provide some brief pointers for caregivers, and the size of any effects that might be promoted is hard to gauge. Research in low resource contexts shows that even very brief interventions can produce surprisingly high levels of change (Mejia *et al*. 2015). It was heartening that parents commented that they valued simply having their difficulties acknowledged, and the significance of this, plus the amount of information needed to bring about change in parent mental health and self-efficacy requires further investigation. Research studies in more secure contexts need to test changes in for example parental sense of competence and changes in child behaviour and emotional adjustment. Establishing the minimal level of provision that can bring about significant change under different circumstances and with different levels of need is an important task for the field, requiring research methods which can maximise information retrieval in complex humanitarian contexts, including for example tsunamis and earthquakes as well as conflicts.

Our aim was to test the feasibility of a novel approach, which proved successful. Testing the effectiveness of materials for use in this context, including both impact on individual families and the transmission of information and sharing of support within wider communities is essential. The methodology we describe provides a means of providing families with very rapid access to information, and researchers with a novel means of collecting data in the field under difficult conditions. Given the scale of need for rapid response in humanitarian emergencies, this approach has the capacity to assist many thousands of people.
